# A framework for sensitivity analysis of decision trees

**DOI:** 10.1007/s10100-017-0479-6

**Published:** 2017-05-24

**Authors:** Bogumił Kamiński, Michał Jakubczyk, Przemysław Szufel

**Affiliations:** SGH Warsaw School of Economics, Al. Niepodległości 162, 02-554 Warsaw, Poland

**Keywords:** Decision trees, Decision optimization, Decision sensitivity

## Abstract

In the paper, we consider sequential decision problems with uncertainty, represented as decision trees. Sensitivity analysis is always a crucial element of decision making and in decision trees it often focuses on probabilities. In the stochastic model considered, the user often has only limited information about the true values of probabilities. We develop a framework for performing sensitivity analysis of optimal strategies accounting for this distributional uncertainty. We design this robust optimization approach in an intuitive and not overly technical way, to make it simple to apply in daily managerial practice. The proposed framework allows for (1) analysis of the stability of the expected-value-maximizing strategy and (2) identification of strategies which are robust with respect to pessimistic/optimistic/mode-favoring perturbations of probabilities. We verify the properties of our approach in two cases: (a) probabilities in a tree are the primitives of the model and can be modified independently; (b) probabilities in a tree reflect some underlying, structural probabilities, and are interrelated. We provide a free software tool implementing the methods described.

## Introduction

Sequentiality and uncertainty are inherent in managerial practice. The former means that managers have to consider multi-staged strategies, encompassing several actions following one another, rather than only a single action; the latter—that a company’s payoffs depend not only on managers’ actions but also on exogenous events (states of the world), which may often be perceived as random from the perspective of the decision maker. The actions and reactions are usually intertwined, further complicating the picture. Decision trees are used as a model that helps in discovering, understanding, and communicating the structure of such decision problems—see Clemen and Reilly (2001) and Waters (2011).

The decision makers are often uncertain about the exact parameters of such trees. In one line of literature, the payoffs are defined imprecisely as intervals, e.g. see Barker and Wilson (2012) and Cao (2014). Another approach that we focus on in the present paper is to assume that a decision maker cannot uniquely assign the probabilities to the possible events (Huntley and Troffaes 2012; Jaffray 2007; Walley 1991). This was dubbed *ambiguity* by Borgonovo and Marinacci (2015); however, other terms are sometimes used (e.g. second-order uncertainty). Importantly, these probabilities are often uncertain in a non-stochastic way, precluding the assignment of any probability distribution (hence, second-order); this was confirmed by our survey among managers taking an MBA course (detailed description of the survey and its results are available at http://bogumilkaminski.pl/pub/mbasurvey.pdf). Such a scenario is a natural setting for adapting ideas from the literature on distributionally robust optimization, see e.g. the work of Delage and Yinyu (2010) and references therein.

In what follows, we assume that if the decision maker knew the probabilities, then she would be willing to base her decision on the expected value principle. Due to non-stochastic distributional uncertainty there is no single expected value; hence, a need for a *sensitivity analysis* (SA) arises, to learn how the output of the decision making process changes when the input is varied—see Saltelli et al. (2009). The importance of a thorough SA is well known and discussed by numerous publications, e.g. Borgonovo and Tarantola (2012). Kouvelis and Yu (2013) point out that uncertainty is a basic structural feature of any business environment and hence should be taken into account in optimal decision making. In particular, uncertainty cannot be replaced by a deterministic model—the optimal solution of a deterministic model is often very different from the optimal solution of a model where uncertainty is present. Kouvelis and Yu (2013) further show that in sequential problems the impact of these uncertainties on decision optimality is even greater than in one-time decisions.

The case when multiple probability distributions can be considered in a decision tree has been previously studied in the literature. Høyland and Wallace (2001) consider assigning a probability density function to each node within a tree for sequential decision making. However, they note that it might be difficult for a user to decide, firstly, what probability density function should be assigned to a particular node and, secondly, how those probabilities should be correlated. Huntley and Troffaes (2012) present several ideas for choice functions, i.e. criteria the decision maker may use to select a subset of strategies. For example, the *maximality* criterion suggests selecting a strategy *X* that is not uniformly worse than some other strategy *Y* (uniformly worse meaning that *Y* offers a greater expected value for all feasible probability distributions). Unfortunately, this criterion may lead to multiple strategies being selected, possibly confusing the decision maker. The solution could be to proceed the other way round: to determine how rich the family of probability distributions may be, in order for the base case strategy to remain optimal; a concept of *admissible interval* (Bhattacharjya and Shachter 2012).

The approach we propose is most suitable when a decision problem is solved once but the optimal strategy is then applied in numerous individual cases. First-order uncertainty can be addressed by calculating the expected value; however, the distributional uncertainty cannot be averaged-out, see the discussion above, as well as in Ben-Tal and Nemirovski (2000), Høyland and Wallace (2001), Huntley and Troffaes (2012) and Kouvelis and Yu (2013). For example, let us consider a bank designing a multi-step debt recovery process. The debt recovery process can be defined and solved for a single debtor, yet the policy developed will be used in numerous cases. Since there may be multiple potentially long paths, the histories of initial implementations will provide only limited information for improving the estimates of the probabilities. Another type of problem that is also addressed in our paper is the situation where many different problems are solved and the long term outcome is what matters, e.g. a capital investor devising financing plans for multiple start-ups. This scenario precludes learning the probabilities from past implementations of the decision. In summary, the setting we propose is valid when the expected value is a natural policy that should be used to select an optimal decision, but it is not reasonable to assume that at the moment of making the decision the decision maker may collect enough data to quantitatively assess the distributional uncertainty of the probabilities in the decision tree.

The contribution of the paper is threefold: (1) a conceptual framework for sensitivity analysis of decision trees; (2) a methodology for performing SA when values in several nodes change simultaneously, and (3) a software implementation that enables practical application of the concepts discussed in the paper. In the following three paragraphs, the contribution is presented in more detail.

Firstly, a single conceptual framework for decision tree sensitivity analysis is created. The framework allows us to conduct threshold proximity SA of decision trees (Nielsen and Jensen 2003), including alternative approaches to that of maximality alone. In particular, the framework is also able to cover the $\varGamma$-maximin criterion (Huntley and Troffaes 2012), under its appropriate parameterization. In doing so, we keep the SA setup simple, e.g. consider only trivial families of probability distribution perturbations, which do not require numerous additional meta-parameters, making it easier to apply by practitioners.

Secondly, the SA methodology proposed in the paper may be conducted simultaneously for multiple parameters in a tree (i.e. the variation in combination, see French (2003)), while the standard approach in the literature is to change parameters one at a time and then inspect the results using a tornado diagram, e.g. Briggs et al. (2006), Briggs et al. (2012), Howard (1988), and Lee et al. (2009). We also show how the results of such SA relate to one another for various types of trees. In particular, we consider non-separable trees, i.e. trees in which probabilities in various parts of the tree are interrelated, as, for instance, to represent the same state of the world. As we show in the paper, the interrelations between the parameters both complicate the analytical approach and can lead to non-intuitive results.

Thirdly, we provide an open source software package that calculates all the concepts defined in the paper—Chondro. The Chondro web page can be accessed at https://github.com/pszufe/chondro/. The software can work on the dictionary representation of decision trees (see the documentation on the software’s home page) as well as being able to open decision trees from SilverDecisions, a software for visual construction of decision trees, available at http://silverdecisions.pl/, which also has decision and value sensitivity analysis functionality.

The paper is organized as follows. In Sect. 2, we introduce a formal model of a decision tree and introduce an important distinction between two types of trees: separable and non-separable. In separable trees, the probabilities in various chance nodes can be changed independently; in non-separable trees, there are constraints defining the relationships between these probabilities. We then present our methods of SA focusing on separable trees in Sect. 3. We discuss how the non-separable case differs in Sect. 4. Section 5 concludes the paper. The proofs of all the remarks have been placed in the Appendix.

## A model of a decision tree

A decision tree is constructed using a directed graph $G=(V,E)$, $E\subset V^2$, with set of nodes *V* (we only consider finite *V*) split into three disjoint sets $V={\mathcal {D}}\cup {\mathcal {C}}\cup {\mathcal {T}}$ of decision, chance, and terminal nodes, respectively. For each edge $e\in E$ we let $e_1\in V$ denote its first element (parent node) and let $e_2\in V$ denote its second element (child node). In further discussion we use the following definition: a directed graph is *weakly connected* if and only if it is possible to reach any node from any other by traversing edges in any direction (irrespectively of their orientation).

**Fig. 1:**
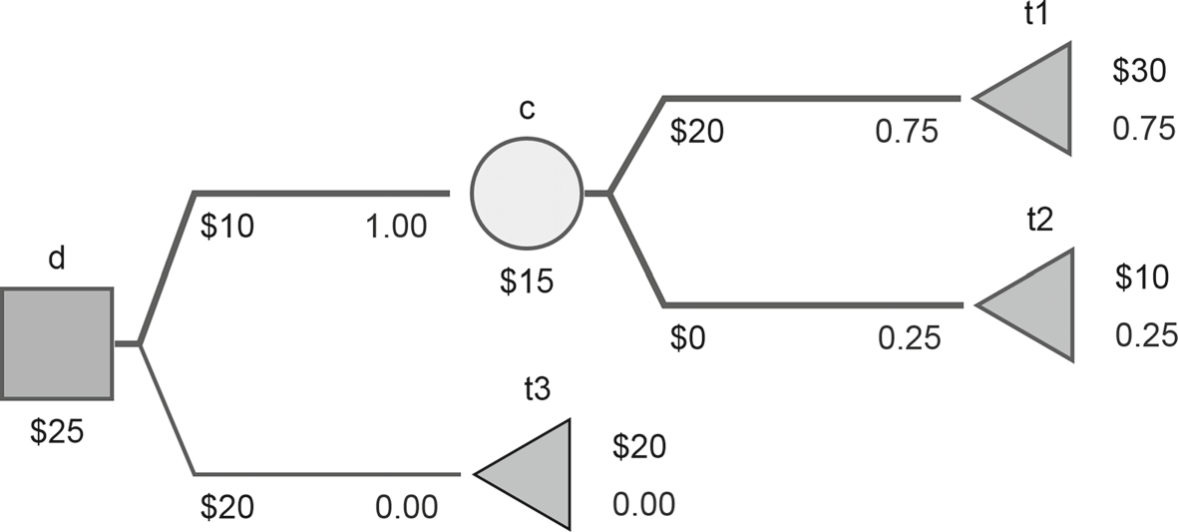
A sample decision tree (all trees drawn using http://silverdecisions.pl/)

Various types of nodes represent different stages of a sequential decision problem. In a decision node, the decision maker selects an action, i.e. one of the edges stemming from this node (one of the edges having the node in question as the parent). In a chance node, one of the edges stemming from it (a reaction) is selected randomly. Terminal nodes represent the end of a sequence of actions/reactions in the decision problem. When drawing a tree, decision nodes are typically represented as squares, chance nodes as circles, and terminal nodes as triangles, usually with the children drawn to the right of their parents. For example, in Fig. 1 we have ${\mathcal {D}}=\{{\textsf {d}}\}$, ${\mathcal {C}}=\{{\textsf {c}}\}$, ${\mathcal {T}}=\{\textsf {t1}, \textsf {t2}, {\textsf {t3}}\}$, and $E=\{({\textsf {d}},{\textsf {c}}),({\textsf {d}},{\textsf {t3}}),({\textsf {c}}, \textsf {t1}),({\textsf {c}},\textsf {t2})\}$.

A decision tree is equipped with two functions: one denoting payoffs, $y:E\rightarrow {\mathbb R}$, and the other denoting probabilities, $p:\{e\in E: e_1\in {\mathcal {C}}\}\rightarrow [0,1]$. With this formalism we make the following assumptions: payoffs are defined for all edges and may follow both actions and reactions; probabilities are defined only for edges stemming from chance nodes. We allow zero probabilities in the general definition of a decision tree, which simplifies the technicalities in subsequent sections. In Fig. 1, we have $y({\textsf {d}},{\textsf {c}})=10$, $y({\textsf {d}},{\textsf {t3}})=20$, $y({\textsf {c}},\textsf {t1})=20$, $y({\textsf {c}},\textsf {t2})=0$ and $p({\textsf {c}},\textsf {t1})=75\%$, $p({\textsf {c}},\textsf {t2})=25\%$.

The decision tree *DT* is a tuple (*G*, *y*, *p*) satisfying the following conditions:

1. there exists $r\in V$ (root) such that $\forall v\in V\setminus \{r\}$ there exists a unique path from *r* to *v*, written as $r\leadsto v$;
2. all and only terminal nodes have no children, i.e. $\forall v\in V:v\in {\mathcal {T}} \Leftrightarrow \lnot \exists e\in E:e_1=v;$
3. $p(\cdot )$ is correctly defined, i.e. $\forall v\in {\mathcal {C}}:\sum _{e\in E, e_1=v}p(e)=1$;

C1 precludes loops in *G* and guarantees that the root is identified. We will let *r*(*DT*) denote the root of *DT*. In Fig. 1, we have $r(DT)={\textsf {d}}$. We will denote all trees where all $p(\cdot )>0$ as *proper trees*; if this condition is not met, we refer to the tree as *improper*.

For a given tree $DT=((V,E),y,p)$, we let *DT*(*v*) denote its subtree rooted in $v\in V$. Formally, $DT(v)=((V',E'),y',p')$ where

- $$V'=\{u\in V:v=u\text { or } v\text { lies on }r(DT)\leadsto u\}$$,
- $$E'=E\cap (V'^2)$$,
- $y'$ and $p'$ are *y* and *p*, respectively, both restricted to $E'$.

In Fig. 1, if we consider $DT({\textsf {c}})$, then $V'=\{{\textsf {c}}, \textsf {t1}, \textsf {t2}\}$, $E'=\{({\textsf {c}},\textsf {t1}),({\textsf {c}},\textsf {t2})\}$, $y({\textsf {c}},\textsf {t1})=20$, $y({\textsf {c}},\textsf {t2})=0$, $p({\textsf {c}},\textsf {t1})=75\%$, and $p({\textsf {c}},\textsf {t2})=25\%$.

The decision maker is allowed to select actions in decision nodes. A *strategy* (policy) in a tree *DT* is defined as a decision function $d:\{e\in E: e_1\in {\mathcal {D}}\}\rightarrow \{0,1\}$, such that $\forall {v\in {\mathcal {D}}}:\, \sum _{e\in E, e_1=v}d(e)=1$. Under this definition the decision maker can only use pure strategies, i.e. explicitly select actions rather than select probabilities and randomize actual actions. A strategy unanimously prescribes an action in every decision node, different strategies (different functions $d(\cdot )$) can, however, be considered as equally good.

We assume that the decision maker maximizes expected payoff, defined as follows:^1^

1 $$\begin{aligned} P({DT},d) = \left\{ \begin{matrix} 0 &{} \quad \text {if} &{} r(DT) \in \mathcal {T}, \\ \sum _{e: e_1=r(DT)} p(e)\left( y(e)+P\left( {DT(e_2)},d\right) \right) &{} \quad \text {if} &{} r(DT) \in \mathcal {C}, \\ \sum _{e: e_1=r(DT)} d(e)\left( y(e)+P\left( {DT(e_2)},d\right) \right) &{} \quad \text {if} &{} r(DT) \in \mathcal {D}. \end{matrix} \right. \end{aligned}$$

Less formally, when being in a terminal node the expected payoff of the remaining actions and reactions amounts to 0. Otherwise, in a decision (chance) node, the expected payoff is defined recursively as the payoff (expected payoff) of the most immediate action (reactions) plus the expected payoff of the subtree (subtrees) we immediately reach.

A strategy *d* maximizing *P*(*DT*, *d*) will be designated as *expected payoff optimal* or *P-optimal*. Under this definition there will usually be many *P*-optimal strategies, because changing *d* for edges down the tree which cannot be reached with a given *d* or *p* (if *p* is zero for some edges, i.e. in an improper tree) does not change *P*(*DT*, *d*). For a given strategy *d* in *DT*, we let *reachable set* (of vertices) to denote a set of vertices of a maximal, weakly connected subgraph of graph $(V,E^*)$ containing *r*(*DT*), where $E^*=\{e\in E:p(e)>0\vee d(e)=1\}$. We will characterize two strategies $d_1$, $d_2$ as *identical*, if their reachable sets are identical. Of course, multiple non-identical strategies may also offer an equal expected payoff and be *P*-optimal.

**Fig. 2:**
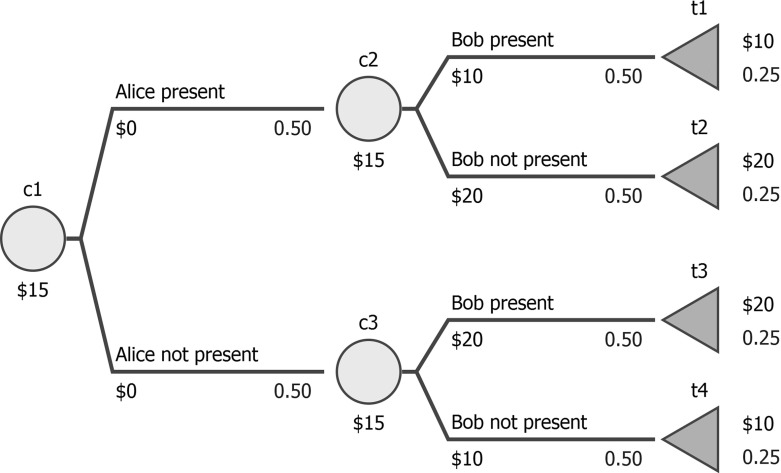
A non-separable decision tree: chance nodes c2 and c3 are interrelated

For future use, we will designate an *almost reachable set* (of vertices) a set of vertices of a maximal, weakly connected subgraph of graph $$(V,E^{**})$$ containing *r*(*DT*), where $$E^{**}=\{e\in E:e_1\in {\mathcal {C}}\vee d(e)=1\}$$. We will categorize two strategies $$d_1$$, $$d_2$$ as *strongly identical* if their almost reachable sets are identical. If two strategies are strongly identical they are identical. Conversely—identical strategies on *G* are strongly identical on a subgraph of *G* where all subtrees starting from edges where $$p(e)=0$$ are removed. Thus, for a proper *DT* we have $$E^*=E^{**}$$, and so the reachable set and almost reachable set coincide for every single strategy. In such a situation two strategies are strongly identical, if they are identical.

Before we discuss the sensitivity analysis methods, we need to introduce the concept of separability proposed by Jeantet and Spanjaard (2009). A decision tree is separable if changing the probabilities in one chance node does not automatically require changing any probabilities in any other chance node (in other words, condition C3 given in the definition of a decision tree in Sect. 2, is sufficient for the probabilities in the tree to be correctly specified). Formally, the set of all allowed probability distributions across all chance nodes is equal to the Cartesian product of the possible probability distributions in every chance node, see Jeantet and Spanjaard (2009).

Often, the probabilities across two or more chance nodes may be interrelated, e.g. entire subtrees may be repeated in the decision tree (coalescence), and two different chance nodes may represent the same uncertain event. In Fig. 2, a host is wondering who will come to her party, and the presence of Alice and Bob is independent. Changing the probability of Bob showing up requires an analogous change in the other chance node, i.e. chance nodes ${\textsf {c2}}$ and $\textsf {c3}$ are interrelated. For future use, observe that Alice and Bob are amiable but dislike each other and the party is only going to be fun if exactly one is present.

Probabilities in different chance nodes may also be interrelated, if they are derived (e.g., using Bayes’ formula) from a common, exogenous parameter. In this case, changing this parameter requires recalculating all the derived probabilities.

If at least two chance nodes are interrelated, we will characterize the entire decision tree as non-separable. Mathematically, non-separability denotes that not all functions *p* are allowed; e.g. in Fig. 2, all such *p* where $p({\textsf {c2}},\textsf {t1})\ne p(\textsf {c3},{\textsf {t3}})$ are forbidden.

When analyzing non-separable trees, we consider a space of *assessed probabilities* which are separable (using terminology presented in Eidsvik et al. (2015)); they need not be directly represented in the tree. The *inferred* probabilities are used in the tree, and they are derived from the assessed probabilities via formulas, possibly linking more than one assessed probability in a given edge. In what follows, we assume that inferred probabilities are continuous in assessed probabilities, which is true when using Bayes’ formula.

As a side note, observe that we could alternatively use a space of assessed (general) *parameters*, i.e. numbers not necessarily restricted to [0, 1] intervals, etc. This change would introduce a qualitative difference between separable and non-separable cases and would require redefining the approach to sensitivity analysis in a way that rendered the two cases less compatible, which is undesirable.

## Sensitivity analysis in separable trees

In this section, we propose several approaches to SA in separable trees. Even though this case may be of limited use in practice, as it requires all the uncertainties to be different along every branch of the tree, it makes it easier to define the ideas first, before proceeding to non-separable trees in the next section. Below, in the first subsection, we focus on the threshold SA, where we analyze how stable a *P*-optimal strategy is. Then, we focus on the scenario SA, and define an optimal strategy for various scenarios of probability perturbation (the range of perturbations for which the currently optimal strategy remains optimal can still be calculated). The notions defined here are then discussed for non-separable trees in Sect. 4.

The decision maker may consider the amount of ambiguity regarding different probabilities as different, and for that reason we introduce an additional function $s:{\mathcal {C}}\rightarrow [0,1]$, representing whether a given node should be subject to sensitivity analysis. In the simplest approach, the decision maker could choose the values of *s*(*c*) from the set $\{0,1\}$, then $s(c)=1$ denotes that *c* should be subject to sensitivity analysis, as the probabilities of events stemming out of this node are not given precisely, and $s(c)=0$ denotes that probabilities are given precisely. Values between 0 and 1 could also be used to denote various degrees of ambiguity (and the formulas below account for this possibility). The choice of the value is subjective and left to the judgment of the decision maker. For instance consider three chance nodes c1, c2 and c3. Chance node c1 represents a coin toss, so the decision maker sets $s(\textsf {c1})=0$ as she assumes that the probabilities are known. Then the decision maker feels that she is twice as certain about the value of probabilities in c2 than c3, so she sets $s({\textsf {c2}})=0.5$ and $s(\textsf {c3})=1$.

### Stability analysis

We define the distance between two decision trees $DT'=((V,E),p',y')$ and $DT=((V,E),p,y)$ to be:

2 $$\begin{aligned} ||DT,DT'||_s=\max _{e\in E, e_1\in {\mathcal {C}}}\frac{|p'(e)-p(e)|}{s(e_1)}. \end{aligned}$$

Observe that the structure of the trees (i.e. (*V*, *E*)) must be identical and that the distance depends on $s(\cdot )$. Payoff functions do not impact the formula and do not need to be identical (typically they will be). In Eq. (2), we take $\frac{0}{0}=0$, effectively forbidding any perturbation for chance nodes with $s(\cdot )=0$, meaning that the decision maker is fully confident with the assigned probabilities. Moreover, in SA we assume that $\exists v\in {\mathcal {C}} : s(v)>0$ (i.e. the the decision maker is uncertain of at least one probability). The above definition is a generalization of the total variation distance for multiple probability distributions (maximum of total variation distances), cf. Tierney (1996).

For further reference, we define a minimum positive sensitivity value:

3 $$\begin{aligned} {\widetilde{s}} = \min \{s(v) : v\in {\mathcal {C}}, s(v) > 0 \} \end{aligned}$$

Observe that in the simple case, where $s(v)\in \{0,1\}$, we have ${\widetilde{s}}=1$.

For a given tree *DT*, sensitivity function $s(\cdot )$, and a *P*-optimal strategy, *d*, we say *d* is $\varepsilon$-stable, if it is also *P*-optimal for any $DT'$ such that $||DT,DT'||_s\le \varepsilon$. In a given tree, *DT*, with sensitivity function, *s*, we then define a *stability index* of a *P*-optimal strategy *d* as:

4 $$\begin{aligned} I(DT,d,s)=\sup \{\varepsilon \in [0,+\infty ]:d\text { is }\varepsilon \text {-stable}\}. \end{aligned}$$

We include *d* explicitly as an argument of $I(\cdot ,\cdot ,\cdot )$ because more than one strategy may be *P*-optimal for *DT*. Observe that Eq. (4) does not yield any results for a non-*P*-optimal *d* (having to calculate $\sup \varnothing$). The definition of *I*(*DT*, *d*, *s*) follows from the following remark, showing that the region of stability is convex.

#### Remark 1

Take $0\le \varepsilon _1\le \varepsilon _2$, a separable decision tree with some sensitivity function $s(\cdot )$, and a *P*-optimal strategy, *d*. If *d* is $\varepsilon _2$-stable, it is also $\varepsilon _1$-stable. If *d* is $\varepsilon _1$-stable for $\varepsilon _1\ge 1/{\widetilde{s}}$, it is also $\varepsilon _2$-stable.

The interpretation of *I*(*DT*, *d*, *s*) is straightforward and should be intuitive even for a non-technical decision maker: if none of the initial probabilities assessed imprecisely change by more than *I*(*DT*, *d*, *s*), then the strategy remains optimal. Thus, the larger the *I*(*DT*, *d*, *s*), the more confident the decision maker may feel about the original *P*-optimal strategy, as a larger deviation is allowed with no consequences for the recommended course of actions.

Based on the properties of *I*(*DT*, *d*, *s*) stated in Remark 1, we can numerically approximate *I*(*DT*, *d*, *s*) using a bisection in $[0,1/{\widetilde{s}}\,]$. For instance, in the simple case where ${\widetilde{s}}=1$ we check the stability for $\varepsilon =\frac{1}{2}$; if *d* is stable, we check $\varepsilon =\frac{3}{4}$, if *d* is not, we check $\varepsilon =\frac{1}{4}$, etc. Verifying stability for a given $\varepsilon$ in separable trees can be done via backward induction. Intuitively, we need to try to modify probabilities (where allowed, i.e. $s(\cdot )>0$) in such a way that *d* is not picked as optimal when solving the tree. That requires worsening the expected payoffs for chance nodes on the optimal path (for the almost reachable set) and improving the payoffs for chance nodes off the optimal path, both in backward induction. Changing payoffs in a single node is done via reallocating probabilities between edges stemming out of this node (cf. Eq. 1) and can be done, for example, using a greedy algorithm or linear programming, as convenient.

A unique *P*-optimal strategy will have a non-trivial region of stability as indicated by the following remark.

#### Remark 2

Take a separable (not necessarily proper) decision tree, *DT*, with some sensitivity function, $s(\cdot )$. Assume all *P*-optimal strategies are strongly identical (have the same almost reachable set). Then, for any *P*-optimal strategy, *d*, we have $I(DT,d,s)>0$.

The stability index is specific for the decision problem as a whole in the following sense.

#### Remark 3

Take a separable proper decision tree, *DT*, with some sensitivity function, $s(\cdot )$. For any two *P*-optimal strategies, $d_1$ and $d_2$, we have $I(DT,d_1,s)=I(DT,d_2,s)$.

Remark 3 will often hold trivially in proper trees in the following sense. If there exist two, non-identical *P*-optimal strategies, $d_1$ and $d_2$, and there is a non-degenerate chance node being in the reachable set of only one of them, then $I(DT,d_1,s)=0=I(DT,d_2,s)$. (A chance node is called non-degenerate, if the expected payoff, cf. equation 1, calculated in this node has a non-zero derivative with respect to probabilities $p(\cdot )$). If there is no such non-degenerate chance node for any pair of non-identical *P*-optimal strategies, then the stability index will be equal and greater than zero. For proper trees, we can then simply let *I*(*DT*, *s*) denote the stability index, meaning it is valid for any *P*-optimal strategy.

The stability index of two strongly identical strategies is also equal for improper trees (not necessarily for two identical strategies: they may start differing when part of a tree starts being reachable after perturbing probabilities). Using Remark 2, we also see that if all *P*-optimal strategies are strongly identical, then this (unique) index will be greater than 0.

Generally, for improper trees there can exist two *P*-optimal strategies with different stability indices. For example, if in Fig. 1 we set $p({\textsf {c}}, \textsf {t1})=0$, $p({\textsf {c}}, \textsf {t2})=1$ and $y({\textsf {d}}, {\textsf {t3}})=10$ and allow perturbation of the probabilities in the chance node c, then both strategies, involving $d({\textsf {d}},{\textsf {t3}})=1$ (lower branch of the tree) and $d({\textsf {d}},{\textsf {c}})=1$ (upper branch), are *P*-optimal, but the stability index of the first is equal to 0 and that of the second is equal to 1.

The managers we surveyed expressed an interest in seeing which strategy is optimal when the perturbation of probabilities is unfavorable and they had strong interest in the most likely outcome of a decision. Regarding the former, observe that unfavorable perturbation may mean different perturbations in a single chance node, depending on which actions are selected in subsequent (farther from the root) nodes. Regarding the latter, we find that simply deleting all edges except for the most likely ones is too extreme and seek to embed this mode-favoring approach into the general framework of maximizing the expected value with modified probabilities. We present our approach in the following subsection.

### Perturbation approach

For a given tree, *DT*, strategy, *d*, a sensitivity function, *s*, and a perturbation bound, $\varepsilon \in [0,+\infty ]$, we define a worst-case-tending expected payoff:

5 $$\begin{aligned} P_{\min }(DT,d,s,\varepsilon )=\min _{\{DT':||DT,DT'||_s\le \varepsilon \}}P(DT',d). \end{aligned}$$

Using a standard maxi-min approach from robust optimization theory (Ben-Tal et al. 2009), we denote strategy *d* as $P_{\min ,\varepsilon }$-optimal, if it maximizes Eq. (5). Obviously, $P_{\min ,0}$-optimality coincides with *P*-optimality. If $\varepsilon \ge 1/{\widetilde{s}}$ then applying $P_{\min ,\varepsilon }$-optimality coincides with a standard Wald (maximin) rule.

Analogously, we can consider a best-case-tending perturbation:

6 $$\begin{aligned} P_{\max }(DT,d,s,\varepsilon )=\max _{\{DT':||DT,DT'||_s\le \varepsilon \}}P(DT',d) \end{aligned}$$

and define a $P_{\max ,\varepsilon }$-optimal strategy as the one that maximizes Eq. (6). Again, $P_{\max ,0}$-optimality coincides with *P*-optimality, and $P_{\max ,1/{\widetilde{s}}}$-optimality coincides with a standard maximax rule.

**Fig. 3:**
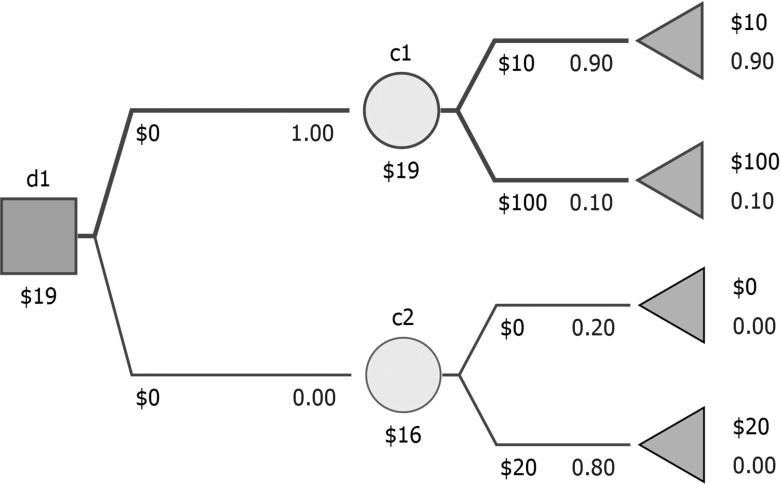
An exemplary separable decision tree where $P_{\min ,\varepsilon }$-optimality regions are not convex

From the computational perspective, for a given $\varepsilon$ we can simply look for a $P_{\min ,\varepsilon }$-optimal and a $P_{\max ,\varepsilon }$-optimal strategy by backward induction, modifying probabilities in chance nodes appropriately. Repeating the calculations for various $\varepsilon$ provides an approximate split of the $[0,1/{\widetilde{s}}\,]$ interval into subintervals in which various strategies are $P_{\min ,\varepsilon }$-optimal and $P_{\max ,\varepsilon }$-optimal. Interestingly, it may happen that a single strategy is $P_{\min ,\varepsilon }$-optimal ($P_{\max ,\varepsilon }$-optimal) for two (or more) subintervals separated by another subinterval. For example, in Fig. 3 the strategy involving $d(\textsf {d1},\textsf {c1})=1$ is optimal for baseline probabilities (displayed in the figure), is not $P_{\min ,0.1}$-optimal (as its expected payoff can fall under such perturbation down to 10), and again is $P_{\min ,1}$-optimal (its expected payoff cannot fall any further).

We now want to introduce a mode-tending perturbation of probabilities, i.e. putting more weight on the most probable events or increasing the contrast between the assigned probabilities—an approach explicitly required by the surveyed managers (in a way, representing being even more certain about the initial assignment of probabilities). Several approaches could be considered here, therefore it is worthwhile explaining why we adopted a specific one by beginning with a discussion of other possibilities. Defining the worst(best)-case-tending can be looked at as finding, for a given node, the set of new probabilities (${\mathbf {x}}=(x_1,\ldots ,x_n)$, probabilities of respective edges stemming from the node), within a ball of radius $\varepsilon$ centered at original probabilities (${{\mathbf {p}}}=(p_1,\ldots ,p_n)$) that minimizes (maximizes) the average payoff of respective subtrees weighted with ${\mathbf {x}}$. One natural idea would be to utilize an analogous approach but, instead, to maximize the average ${{\mathbf {p}}}$ weighted with ${\mathbf {x}}$, which would enforce putting even more emphasis on likely events (we would tend to increase those components of ${\mathbf {x}}$ which correspond to the large components of ${{\mathbf {p}}}$). The disadvantage of this approach is that reversing it (minimizing the average) leads not to assigning equal probabilities to all the events (within respective chance nodes, which we would consider as natural), but to selecting the least-likely events, which is an odd scenario.

Another approach would be to use the entropy of ${\mathbf {x}}$. That works nicely for maximization (leading to Laplacean, equal probabilities), but does not unequivocally select one set of probabilities when minimizing entropy. That is why we decided to use divergence (Kullback and Leibler 1951), given by the formula

7 $$\begin{aligned} D_{KL}(P||Q) = \sum _{i\in A} P(i)\log \left( \frac{P(i)}{Q(i)}\right) , \end{aligned}$$

where *P* and *Q* are discrete probability distributions having the same domain *A*. It is a measure of the non-symmetric difference between two probability distributions. For various ${\mathbf {x}}$ with a given entropy, we want to select the one that is closest to the original ${{\mathbf {p}}}$, i.e. minimizes $D_{KL}({\mathbf {x}}||{{\mathbf {p}}})$. It can be written equivalently as the following optimization task for ${\mathbf {x}}$ with parameter $\theta$:

8 $$\begin{aligned} \begin{aligned}&\text {minimize}\quad \quad D_{KL}({\mathbf {x}}||{{\mathbf {p}}})\\&\text {subject to: } \,\,\quad D_{KL}({\mathbf {x}}||{\mathbf {u}})=\theta \,\wedge \, {\mathbf {x}}\ge \mathbf (0) \,\wedge \, \sum _{i=1}^n x_i=1, \end{aligned} \end{aligned}$$

where ${\mathbf {u}}=(\frac{1}{n},\ldots ,\frac{1}{n})$ (of length *n*). Taking $\theta =0$ yields equal probabilities (Laplace case), and that is why using $D_{KL}({\mathbf {x}}||{\mathbf {u}})$ in a constraint is more illustrative than entropy (while equivalent). Increasing $\theta$ leads to considering distributions more and more concentrated in single points. Solving this task yields a convenient looking formula, presented in the following remark.

#### Remark 4

The solution of the optimization problem (8) for various values of $\theta$ yields ${\mathbf {x}}$ changing along the path given by the following soft-max formula with parameter $\gamma \in [0,+\infty [$:

9 $$\begin{aligned} x_i=\frac{p_i^\gamma }{\sum _{j=1}^{n} p_j^\gamma }. \end{aligned}$$

Using $\gamma$ is more convenient than using $\theta$: $\gamma =0$ implies equating all probabilities in ${\mathbf {x}}$ (and corresponds to $\theta =0$), $\gamma =1$ implies using original probabilities ${\mathbf {x}}={{\mathbf {p}}}$ (corresponds to $\theta =D_{KL}({{\mathbf {p}}}||{\mathbf {u}})$), and for $\gamma \rightarrow +\infty$ the probabilities in ${\mathbf {x}}$ concentrate in a mode (modes) of ${{\mathbf {p}}}$ (all $x_i$ corresponding to probabilities $p_i$ less than $\max p_i$ tend to 0, and all the remaining ones tend to equal positive values). Simple algebraic manipulations show that $dx_i/d\gamma |_{\gamma =1}>0$ if and only if $p_i>\prod _{j=1}^np_j^{p_j}$, i.e. a geometric mean of $p_i$ weighted by $p_i$. In short, if $p_i$ is large, it gets larger; and if it is small, it gets smaller.

**Fig. 4:**
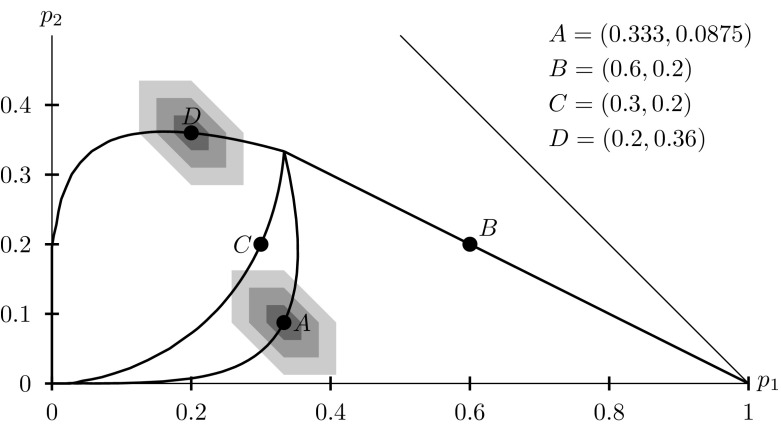
The lower part of a Machina triangle with paths for four various probability distributions (*dots*) perturbed with a softmax formula. $\varepsilon$-balls shaded in *gray* for two initial distributions

In Fig. 4, we illustrated paths of ${\mathbf {x}}$ (thick lines) for various starting points (${{\mathbf {p}}}$, four thick dots). In the figure, we can see two coordinates of a three-element vector of probabilities, with the third being residual value, a well known technique called a Machina triangle (Machina 1987), we only show the lower half of it). For $\gamma =0$, all the paths meet in $(\frac{1}{3},\frac{1}{3})$; for $\gamma \rightarrow +\infty$, they wander towards vertices of the triangle. As can be seen, the paths may be straight segments but may also involve non-monotonicity, both in the $\gamma \in [0,1]$ and in $\gamma \in [1,+\infty [$ part. We denoted with shaded regions various $\varepsilon$-balls around two original probabilities (*A* and *D*).

We can now define $P_{\text {mode},\varepsilon }$-optimality. For a given $\varepsilon \ge 0$ we transform in each chance node the probabilities according to Eq. (9) for as large $\gamma$ as possible while $||DT,DT'||_s\le \varepsilon$. Such $\gamma$ can be found by means of any one-dimensional root-finding algorithm, since increasing $\gamma$ increases $\max _i|x_i-p_i|$. Observe that in each chance node $\gamma$ is selected independently. For these perturbed probabilities, we select the expected payoff maximizing decision, denoting it as $P_{\text {mode},\varepsilon }$-optimal. Repeating the calculations for various $\varepsilon$ provides an approximate split of the $[0,1/{\widetilde{s}}\,]$ interval into subintervals in which various decisions are $P_{\text {mode},\varepsilon }$-optimal.

The notions of stability and $P_{\cdot ,\varepsilon }$-optimality can be linked.

#### Remark 5

Take a separable tree, *DT*, with a sensitivity function, $s(\cdot )$. If *d* is *P*-optimal, then it is also $P_{\min ,\varepsilon }$-optimal, $P_{\max ,\varepsilon }$-optimal, and $P_{\text {mode},\varepsilon }$-optimal for any $\varepsilon \le I(DT, d, s)$.

**Fig. 5:**
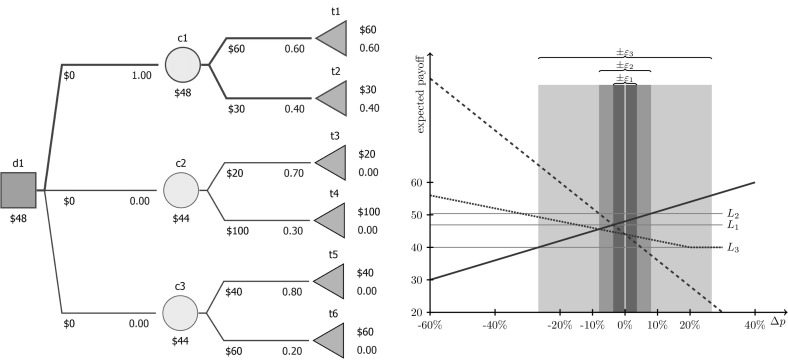
An exemplary decision tree (*above*) with sensitivity analysis (*below*) for three strategies (going to $\textsf {c1}$, ${\textsf {c2}}$, and $\textsf {c3}$ represented with *thick solid*, *dashed*, and *dotted line*, respectively). The *horizontal axis* denotes the deviation in the probability of going to the upper node ($\textsf {t1}$, ${\textsf {t3}}$, and $\textsf {t5}$, respectively) independently for each strategy. $\textsf {c1}$ is optimal for *baseline* probabilities, shaded regions illustrate boundaries for stability (*dark*), $P_{\max ,\varepsilon }$-optimality (*medium*), and $P_{\min ,\varepsilon }$-optimality (*light*). It is $P_{\text {mode},\varepsilon }$-optimal for all the deviations (mode-tending requires maximizing ${\varDelta {p}}$). *Gray horizontal lines* drawn to help see where expected payoffs equate (observe that the expected payoff of the decision to choose c3 temains constant for ${\varDelta {p}}>20\%$ as probability of t6 is equal to $20\%$). $\varepsilon _1\approx 3.63\%$, $\varepsilon _2=8\%$, and $\varepsilon _3\approx 26.67\%$

The relation between stability and $P_{\cdot ,\varepsilon }$-optimality is illustrated in Fig. 5. The left part presents an exemplary tree with three strategies $d_1$, $d_2$, and $d_3$ (setting, respectively, $d(\textsf {d1},\textsf {c1})=1$, $d(\textsf {d1},{\textsf {c2}})=1$, and $d(\textsf {d1},\textsf {c3})=1$) and baseline probabilities. $d_1$ is *P*-optimal. The right part presents the impact of modifying probabilities on the expected payoff of these three strategies. The horizontal axis presents the deviation in the probability of selecting the upper edge (leading to t1, t3, and t5, respectively); the probability for the lower edge changes residually. We independently set the individual deviations for $d_1$, $d_2$, and $d_3$, but we decide jointly on the range of feasible deviations (the width of a shaded region). Expected payoffs are denoted with solid, dashed, and dotted lines for $d_1$, $d_2$, and $d_3$, respectively. If we allow the probabilities to vary in the range of $\varepsilon _1\approx 3.63\%$, then $d_1$ remains *P*-optimal, while beyond this range it may start losing to $d_2$ (when $p(\textsf {c1},\textsf {t1})$ and $p({\textsf {c2}},{\textsf {t3}})$ are decreased, marked with a thin horizontal line). If the decision maker is confident that the initial probabilities are imprecise by no more than 3.63%, then $d_1$ is definitely a good choice.

If the probabilities may vary by more than 3.63%, then $d_1$ may not be *P*-optimal. Then, the decision maker may prefer to make a safe choice, i.e. select a strategy that offers the greatest expected payoff in the case of the most unfavorable perturbation. Obviously, for perturbations within $\varepsilon _1$, $d_1$ is such a strategy (cf. Remark 5). As Fig. 5 shows, for deviations smaller than $\varepsilon _3\approx 26.67\%$, $d_1$ remains $P_{\min ,\varepsilon }$-optimal. Only when we allow a larger deviation, may the possible expected payoff of $d_1$ be worse than the worst possible expected payoff of $d_3$; hence, $d_1$ ceases to be the safest choice.

If we think in terms of the most likely outcomes, then no matter which deviation is allowed, $d_1$ remains $P_{\text {mode},\varepsilon }$-optimal, because mode-tending means increasing the probability of a greater payoff for $d_1$ and of a smaller payoff for $d_2$ and $d_3$ (in all cases it denotes moving to the right in Fig. 5).

One more remark is due. The results of the stability analysis and $P_{\cdot ,\varepsilon }$-optimality depend strongly on how the decision problem is structured. For instance, we can split a single edge stemming out of a chance node into two edges and assign them half of the original probability. Nothing has changed in terms of the base case representation of the problem. However, a given $\varepsilon$ now effectively allows for twice as large a variation in the probability of the event (before split), and so the results of all the analyses may change. On the one hand, this may be perceived as a disadvantage of the proposed methods but, on the other hand, we would argue that selecting a particular way of representing the problem apparently provides insight into how a decision maker perceives the distinct uncertainties, events, and imprecise probabilities. It is therefore not surprising that changes in perception should be reflected in changes in the results of SA.

## Sensitivity analysis in non-separable trees

In non-separable trees, the probabilities in various chance nodes cannot be perturbed independently, which represents a challenge for the algorithms presented above. Backward induction would not yield the correct results, e.g. in Fig. 2, when assessing $P_{\min ,\varepsilon }$-optimality (assuming this tree is a part of some decision), backward induction would increase $p({\textsf {c2}},\textsf {t1})$ (i.e. Bob present) in ${\textsf {c2}}$ and at the same time increase $p(\textsf {c3},\textsf {t4})$ (i.e. Bob not present) in $\textsf {c3}$.

As mentioned in the last part of Sect. 2, this could be modeled in terms of some additional restrictions on the $p(\cdot )$ function but we find it more intuitive to assume that the probabilities reflected in the tree are derived from some more primitive probabilities, *assessed probabilities* (denoted with capital *P*), which themselves are separable and represent discrete distributions. In the case illustrated in Fig. 2, this assumption would imply using two assessed probabilities: $P(\text {Alice present})$ and $P(\text {Alice not present})$ (*P*(*A*) and ($P(\lnot A)$ in short, used to define $p(\cdot )$ in $\textsf {c1}$) and $P(\text {Bob present})$ and $P(\text {Bob not present})$ (*P*(*B*) and $P(\lnot B)$, similarly used to define $p(\cdot )$ in ${\textsf {c2}}$ and $\textsf {c3}$).

We now suggest defining $s(\cdot )$ and calculating $\varepsilon$-deviations in the space of assessed probabilities. This approach requires redefining the notions introduced in Sect. 3 into the space of assessed probabilities. Hence, we require that assessed values are indeed probabilities, rather than arbitrary parameters and that they represent discrete distributions (in a sense, *virtual* chance nodes). For example, for the distribution function $F_A$, representing the fact of Alice being present or not, we have two assessed probabilities *P*(*A*) and $P(\lnot A)$. For concrete values of $s(F_A)$ and $\varepsilon$, we have the constraint that neither of these probabilities in SA can diverge from the initial values by more than $s(F_A)\varepsilon$.

A new problem arises in the non-separable case, since the expected payoff is in general no longer convex in the space of assessed probabilities. In our example from Fig. 2 it amounts to 15 for $P(A)=\frac{1}{2}$, $P(B)=\frac{1}{2}$, and to 20 for $P(A)=1$, $P(B)=0$ and $P(A)=0$, $P(B)=1$, and to 10 for $P(A)=1$, $P(B)=1$ and $P(A)=0$, $P(B)=0$. Thus, if we want to find a pessimistic or an optimistic evaluation of a given strategy, d, and $\varepsilon$, we have to use an algorithm that takes into account that there might be multiple local minima of the expected payoff. In our implementation, we use a simple grid search over assessed probabilities but for large trees a more efficient algorithm might be needed (e.g. a genetic algorithm). In consequence, looking for a $P_{\min ,\varepsilon }$-optimal or a $P_{\max ,\varepsilon }$-optimal strategy is more difficult than in the separable case: an exhaustive search over all strategies in a decision tree is required and for a single considered strategy a global optimization has to be performed. Determining the $P_{\text {mode},\varepsilon }$-optimal strategy remains straightforward: it suffices to perturb assessed probabilities using the softmax rule and to calculate the optimal strategy in the modified tree.

**Fig. 6:**
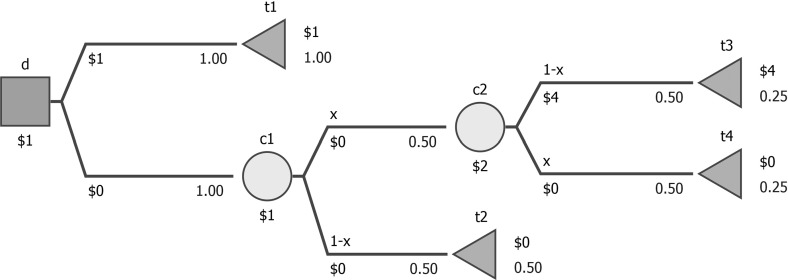
A sample non-separable decision tree for which Remark 3 does not hold (*x* is an assessed probability initially set to 0.5)

Remarks 1, 2, and  5 remain valid in the non-separable case, and the proofs follow the same lines. Observe that only Remark  2 requires the assumption that mapping between assessed and inferred probabilities is continuous. Remark  3 is unfortunately not true, see Fig.  6 for an example. In the decision tree, *x* is an assessed probability, initially set to 0.5. The probabilities in chance nodes c1 and c2 are derived from *x*. Two strategies ($d(\textit{d},\textit{t1})=1$ and $d(\textit{d},\textit{c1})=1$) are *P*-optimal, but the former remains so for any perturbation of *x* (stability index equal to 1), and the latter ceases being *P*-optimal for any perturbation (stability index equal to 0), as $4x(1-x)<1$ for $x\ne 0.5$.

Let us examine how the proposed methodology works in a more complicated, non-separable case. Consider an investor owning a plot of land, possibly (a priori probability amounting to 70%) hiding shale gas layers. The plot can be sold immediately (800, all prices in $’000). The investor can build a gas extraction unit for a cost of 300. If gas is found, the profit will amount to 2,500 (if not, there will be no profit, and no possibility of selling the land). Geological tests can be performed for a cost of 50, and will produce either a positive or a negative signal. The sensitivity amounts to 90%, and the specificity amounts to 70%. The installation can be built after the test or the land may be sold for 1,000 (600) after a positive (negative) test result.

As mentioned in Sect. 2, representing this problem from the decision maker’s perspective requires transforming the above probabilities. Observe that the probabilities, as given in the text above, are not logically interrelated, so they can be modified without forcing other probabilities to be changed also. Thus, they form the assessed probabilities, namely: $P(\text {gas})$, $\text {sensitivity}$, and $\text {specificity}$. All three probabilities represent binary distributions; in order to simplify the notation further, we propose performing a sensitivity analysis of these probabilities (however, it should be remembered that the complements of these probabilities are also assessed probabilities). We keep in mind that in general we perform the sensitivity analysis on discrete distributions—this would be important, if we had more than two possible outcomes in a distribution represented by assessed probabilities.

**Fig. 7:**
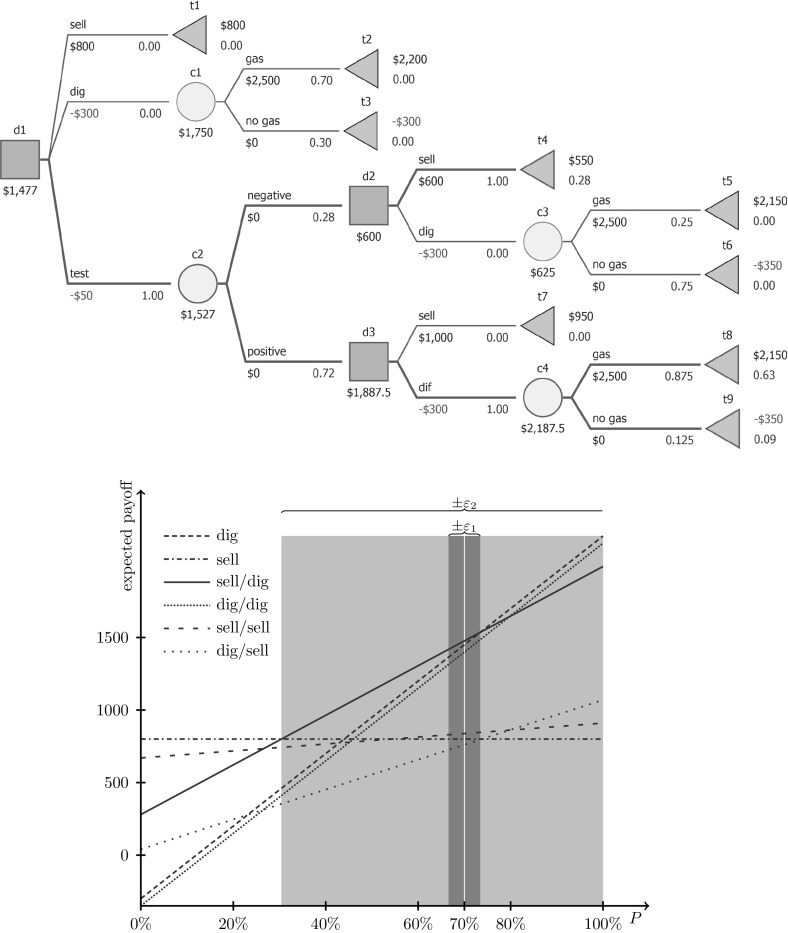
A non-separable decision tree for the gas problem (*above*) with sensitivity analysis (*below*) for the assessed probability $P(\text {gas})$ with fixed sensitivity and specificity. The reachable set for *P*-optimal strategies in the tree (*above*) are marked with thicker edges. The strategies are represented with lines and depend on the assessed probability. The strategy *sell for negative test)/dig otherwise* (*solid thick line*) is optimal for baseline probability $P(\text {gas})=0.7$. The *dark shaded* regions illustrate the boundary for stability $\varepsilon _1\approx 3.42\%$. For the mode $P_{\text {mode},\varepsilon }$-optimality and $P_{\max ,\varepsilon }$-optimality perturbation the same epsilon value is valid; for larger $\varepsilon$ the optimal strategy changes to *dig* (see the *right side of the dark area*). The $P_{\min ,\varepsilon }$-optimality perturbation does not change the optimal strategy up to $\varepsilon _2\approx 39.59\%$; for larger $\varepsilon$ the optimal strategy is to *sell* (see the *left side of the light-gray area*)

The structure of actions and reactions available to the decision maker requires using another set of probabilities, as presented in Fig. 7. The tree-probabilities are linked to the assessed ones via the following formulas:

$$\begin{aligned} P(\text {pos.~test})&=\text {Sensitivity}\times P(\text {gas})+(1-\text {specificity})\times P(\text {no gas}),\\ P(\text {neg.~test})&=(1-\text {sensitivity})\times P(\text {gas})+\text {specificity}\times P(\text {no gas}),\\ P(\text {gas} | \text {pos.~test})&=\frac{\text {Sensitivity}\times P(\text {gas})}{P(\text {pos.~test})},\\ P(\text {gas} | \text {neg.~test})&=\frac{(1-\text {sensitivity})\times P(\text {gas})}{P(\text {neg.~test})}, \end{aligned}$$

which are continuous functions of assessed probabilities.

It is *P*-optimal to perform the test and build the gas extraction system only when the result is positive (sell the land otherwise). On the bottom Fig. 7 we can see the stability and perturbation analysis assuming that sensitivity and specificity values are known exactly (i.e. $s(\text {specificity})=0$ and $s(\text {sensitivity})=0$). The dark shaded regions illustrate the boundary for stability $\varepsilon _1\approx 3.42\%$. For the mode $P_{\text {mode},\varepsilon }$-optimality and $P_{\max ,\varepsilon }$-optimality perturbation the same epsilon value is valid; for larger $\varepsilon$ the optimal strategy changes to *dig* (the right side of the dark area). The $P_{\min ,\varepsilon }$-optimality perturbation does not change the optimal strategy up to $\varepsilon _2\approx 39.59\%$; for larger $\varepsilon$ the optimal strategy is to *sell* (the left side of the light-gray area).

Now let us assume that the investor does not know the exact values of sensitivity and specificity for the existence of gas test, although this uncertainty is quite low. Specifically, we assume $s(P(\text {gas}))=1$, $s(\text {specificity})=0.1$, and $s(\text {sensitivity})=0.1$. The stability of the base optimal strategy (*test: sell if negative, dig if positive*) is $2.90\%$. It is natural that the stability has decreased in comparison to the previous scenario as we allow sensitivity and specificity to be perturbed and they do not affect the *immediately dig* strategy. $P_{\text {mode},\varepsilon }$ and $P_{\max ,\varepsilon }$ perturbations in the range $\varepsilon \in [0\%, 4.16\%[$ do not change the *P*-optimal strategy while for the $\varepsilon \in ]4.16\%,100\%]$ the optimal strategy is to *immediately dig*. Again, this result (wider interval for base optimal strategy) might have been expected, since a favorable perturbation of sensitivity and specificity increases their values and thus makes the base optimal strategy more attractive. Finally, for the $P_{\min ,\varepsilon }$ perturbation in the range $\varepsilon \in [0\%, 37.14\%[$, the optimal strategy does not change while for $\varepsilon \in ]37.14\%, 100\%]$ the optimal strategy is to *immediately sell*. Similarly to previous perturbation methods, the decrease of the interval width for the base decision follows the fact that sensitivity and specificity do not affect the *immediately sell* strategy.

## Concluding remarks

In the paper, we presented a framework for performing SA in decision trees when some probabilities are not known precisely. In this framework, we tried to encompass what managers declared to be of interest when analyzing decision problems with uncertainty: verifying the impact of modifying probabilities on the decision remaining optimal, thinking in terms of unfavorable/favorable perturbations, or thinking in terms of most likely outcomes. All these approaches can be unified in a single model, calculated in the software we provide, and illustrated for sample cases (see Fig. 5). We found that it is crucial whether the probabilities in the tree can be set independently between various chance nodes, i.e. whether a tree is separable. If not, then a more complicated approach needs to be taken to define the model, and additionally more complex algorithms to perform SA need to be used.

Our approach to SA allows the decision maker to examine the advantages and disadvantages of the available decision alternatives from several angles. Figure 5 nicely illustrates how various approaches to SA can yield different answers. As with all the decision support tools—it is the decision maker who needs to make the final decision and is responsible for it.

As mentioned in the introduction, the methods we suggest can be linked to ideas discussed, e.g. by Huntley and Troffaes (2012): $\varGamma$-maximin, maximality/E-admissibility, and interval dominance. Hence, $P_{\min }$-optimality is directly equivalent to $\varGamma$-maximin. Still, the difference is that rather than treating the set of probability distributions as given exogenously, we build it endogenously instead, verifying how large it can be (in terms of Eq. (2), around the baseline probabilities) for the base-case *P*-optimal strategy to be $P_{\min }$-optimal.

The stability index defines a set of probability distributions in which the *P*-optimal strategy is the only maximal and the only E-admissible one. Again, in our approach we do not use maximality/E-admissibility to make a choice for a given set of probabilities but instead define the strength of the *P*-optimal strategy by looking at how imprecise the original probabilities can be for this strategy to remain the only maximal/E-admissible one. In our approach, the difference between maximality and E-admissibility is inconsequential.

**Fig. 8:**
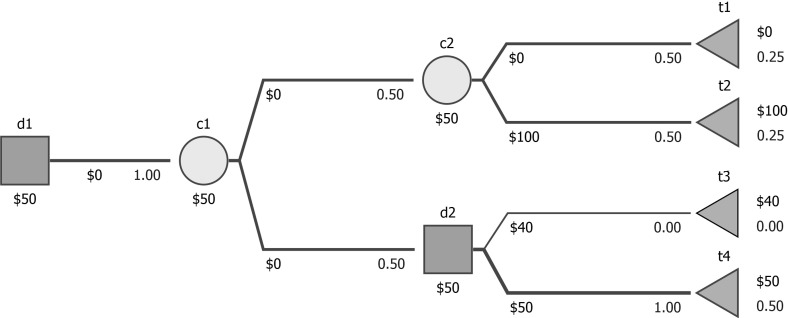
An example: interval dominance does not hold within the stability region of a *P*-optimal decision (to go down from d2)

The situation is more complicated for interval dominance. The *P*-optimal strategy is surely not interval dominant beyond the stability index. For separable trees, the *P*-optimal strategy will also be interval-dominant within the region defined by the stability index if it does not have a common chance node with another decision. Otherwise, the *P*-optimal strategy may not be interval dominant even within the region defined by the stability index, see Fig. 8 (selecting t4 in d2 is obviously *P*-optimal and it is 1-stable, while it does not interval-dominate selecting t3 when probabilities can by changed by 0.1). That suggests that our approach is significantly different from interval dominance.

The ideas presented in the present paper also relate to the information-gap theory proposed and developed by Ben-Haim (2001), where one measures for each strategy how much uncertainty is allowed (i.e., how large a deviation of model parameters is allowed) for the considered strategies to definitely offer a pay-off greater than some assumed minimal level (i.e. the robustness), or in another approach: how much uncertainty is needed to make it possible for the strategies to offer some assumed desired outcome (i.e. the opportuneness). Both approaches, ours and Ben-Haim’s, are local, i.e., there is some baseline value of uncertain parameters from which the deviations are considered (and not simply a family of possible parameterizations is considered). Also, in both approaches no probability distributions are assigned to the deviations. Lastly, we consider both the unfavorable and favorable deviations, as analogs of robustness and opportuneness, respectively. Nevertheless, there are important differences. Firstly, we apply our ideas specifically to decision trees; hence, the contribution of the present paper also lies in how the ideas are implemented in that particular context. Secondly, the line of thinking in the information-gap approach goes from the desired outcome (e.g., minimal required outcome) to the amount of uncertainty (guaranteeing this threshold is exceeded), while we treat the amount of ambiguity related to parameters as the starting point and proceed towards the recommended strategy (and, e.g., the minimal guaranteed outcome). We find treating the ambiguity as primitive and the resulting satisfaction as an outcome to be more intuitive and to follow the cause-effect path. Thirdly, we also present our own additional extensions to the SA (e.g., mode favoring deviations).

There are some limitations to the present study and pertinent ideas for further research. We used a very simple definition of the distance between two trees, cf. Eq. (2). This metric allows multiple probabilities to differ simultaneously from their baseline values, not aggregating individual differences to reflect that overall the set of probabilities changed substantially (as, for example, the sum of absolute deviations would do). We would maintain that as long as the probabilities in various chance nodes are unrelated (i.e. we consider separable trees), this is a desired feature. The decision maker can express the degree of imprecision (and possibly differentiate it between various nodes with $s(\cdot )$) but this imprecision can simultaneously affect several chance nodes: being more incorrect in one chance node does not increase the precision of knowing the true probabilities in some other chance node. Moreover, such a definition is simple and intuitive to understand for the decision makers.

We only calculate the stability index of the optimal strategy. At first glance, it may be of interest to know how stable the second-optimal strategy is, especially if the stability index of the optimal strategy is small. Should the stability index of the second best one (somehow defined) be large, we might be tempted to select it, since it looks robust, even if only *second*-optimal. For example, assume the stability index for the first-optimal $d_1$ equals 0.02 (i.e. 2 pp), and for the second optimal $d_2$ it amounts to as much as 0.3 (i.e. 30 pp). The true interpretation, however, would be the following. If baseline probabilities sufficiently approximate the true ones (within 2 pp), then $d_1$ is sure to maximize the expected payoff (be optimal). If the imprecision is larger than 2 pp (but smaller than 30 pp), then $d_1$ might not be optimal ($d_2$ might be); yet $d_1$ might still be optimal for deviations larger than 2 pp! These stability indices guarantee that if the deviation is within 30 pp, then either $d_1$ or $d_2$ is optimal, but there is still no reason to favor $d_2$ over $d_1$. This is also related to the fact that in our research we focused on *decision sensitivity* (Nielsen and Jensen 2003), and not value sensitivity, i.e. we analyze when the optimal strategy changes with varying input, not by how much the payoff is reduced.

If the decision maker is concerned with a possibly greater negative impact of perturbations on one strategy and wants to select a safe (even if not optimal) strategy, then $P_{\min ,\varepsilon }$-optimality is the appropriate concept. Figure 5 nicely shows that even if the optimal strategy has a relatively small stability index, it is still very safe for large deviations, i.e., it offers the highest guaranteed (worst-case) expected value. Observe that putting greater weight on less favorable outcomes (for each strategy separately) may also be interesting when no ambiguity is present. It may be taken to represent risk aversion somewhat similarly to rank-dependent utility models, in which we re-weight the probabilities, overweighting the extremely unfavorable and underweighting the extremely favorable ones—see Quiggin (1982). In the case of $P_{\min ,\varepsilon }$-optimality, for sufficiently small $\varepsilon$ we only reweight two single outcomes (the most unfavorable and the most favorable one) in a given chance node, but this reweighting builds up over multiple chance nodes in a non-trivial way (e.g. perturbed probabilities in one chance node being multiplied by perturbed probabilities in another chance node). Another approach to model risk aversion would be to transform payoffs to von Neumann–Morgenstern utilities (we would have to attribute the utilities only up to the edges just before the terminal nodes or account for the fact that the marginal utility is diminishing as payoffs aggregate along the paths in the tree).

## Figures and Tables

**Fig. 9:**
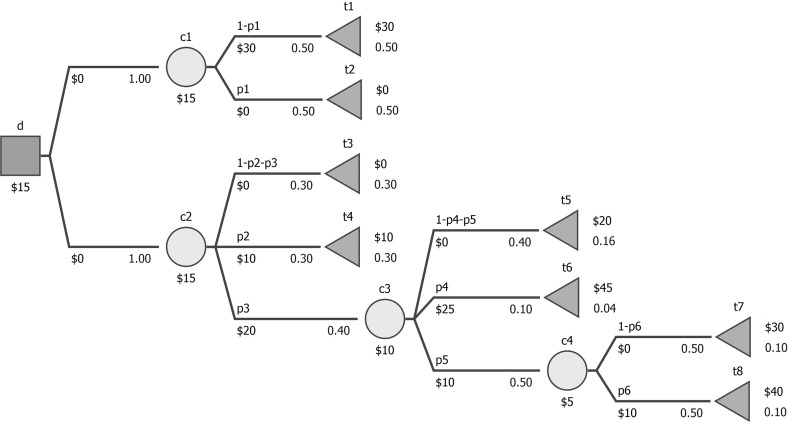
Illustration to the proof of Remark  3

## Appendix: Proofs

### Proof (of Remark 1)

The first part of the remark follows from $\{DT':||DT,DT'||_s\le \varepsilon _1\}$ being a subset of $\{DT':||DT,DT'||_s\le \varepsilon _2\}$. The second part follows, as $\{DT':||DT,DT'||_s\le 1/{\widetilde{s}}\,\}$ contains all possible $p(\cdot )$ parameterizations of the considered tree (any change of probabilities is within the allowed limits). This second part is useful in numerical procedures to determine *I*(*DT*, *d*, *s*) effectively by bisecting over the interval $[0,1/{\widetilde{s}}\,]$. $\square$

### Proof (of Remark 2)

Take any *P*-optimal strategy *d*. Let $d_1$, $d_2$, ..., $d_n$ denote strategies non-strongly-identical to *d* (a finite set for finite trees). It must be $P(DT,d_i)<P(DT,d)$ for $i\in \{1,2,\ldots ,n\}$, as *d* is a unique $P(\cdot )$-maximizing strategy (up to being strongly identical). Observe that

1. $P(DT,d,s)-\max \{P(DT,d_1,s),\ldots ,P(DT,d_n,s)\}$ is a continuous function of $p(\cdot )$,
2. changes in $p(\cdot )$ do not change the almost reachable set of any strategy, and so the set of strategies strongly identical to *d* is left unchanged,
3. strongly identical strategies have the same expected payoff (for a given $p(\cdot )$).

Observations 1–3 give the required result. Properness is not used. $\square$

### Proof (of Remark 3)

We follow the proof by contradiction.

Take two *P*-optimal strategies, $d_1$ and $d_2$. Obviously, $P(DT,d_1)-P(DT,d_2)=0$. Assume without loss of generality $I(DT,d_1,s)>I(DT,d_2,s)$. Then $I(DT,d_1,s)>0$, and so there exists a tree $DT'$ with perturbed probabilities in which $d_1$ is *P*-optimal while $d_2$ is not. Hence, in $DT'$ we have $P(DT',d_1)-P(DT',d_2)>0$. Notice, additionally, that the expression $P(DT',d_1)-P(DT',d_2)$ is continuous in the probabilities of the tree for any probabilities (e.g. those of *DT*, $DT'$).

We will show that there exists a tree $DT^*$ arbitrarily close to *DT* (in the sense of Eq. 2) such that $P(DT^*,d_1)-P(DT^*,d_2)<0$, which yields a contradiction as it would forbid $I(DT,d_1,s)>0$. Note, that for that purpose it suffices to show that *DT* (more precisely: values of probabilities for *DT*) is not a local extremum of $\scriptstyle \varDelta (X)\overset{\text {def}}{=}P(X,d_1)-P(X,d_2)$, as then in the vicinity of *DT* we must also have $\varDelta (\cdot )<0$. We will verify that *DT* is not an extremum by inspecting the Hessian, *H*, of $\varDelta (X)$ at *DT*.

$P(X,d_1)$ can be be written as a sum of products of probabilities of reaching respective terminal nodes (products of probabilities along the paths) and total payoffs for these nodes (sum of payoffs along the paths). We can additionally in each chance node express a single probability as a residual value. Thus, $\varDelta (X)$ is also a sum of products of this type. See Fig. 9 for illustration. Decision setting $d(\textit{d},\textit{c1})=1$, denoted $d_1$, offers the expected payoff equal to $30(1-p_1)$, while setting $d(\textit{d},\textit{c2})=1$, denoted $d_2$, offers the following expected payoff:

$$\begin{aligned} 10p_2+20p_3(1-p_4-p_5)+45p_3p_4+30p_3p_5(1-p_6)+40p_3p_5p_6, \end{aligned}$$

which simplifies to $10p_2+20p_3+25p_3p_4+10p_3p_5+10p_3p_5p_6$. Then the difference in payoffs is given as $30-30p_1-10p_2-20p_3-25p_3p_4-10p_3p_5-10p_3p_5p_6$.

Due to the properness of the tree and the fact that one probability in every chance node was left out as a residual we can at least to some extent freely perturb all the parameters in the above-defined expression. Also note that each single probability is either absent or present in the first power. Therefore, for any single probability *p*, we have $\partial ^2\varDelta /\partial p^2=0$, and so the main diagonal of *H* only contains zeros. Now consider any two probabilities, denoted $p_A$ and $p_B$ to avoid confusion with the exemplary parameters of Fig. 9. If $\partial ^2\varDelta /\partial p_Ap_B\ne 0$ at *DT*, then, by Sylvester’s criterion, *H* is non-definite (as after reorganizing probabilities the second principal minor is negative), and so $\varDelta (X)$ does not have an extremum at *DT*, which completes the proof. We are left with the case that all $\partial ^2\varDelta /\partial p_Ap_B=0$ at *DT*. In the next step we will show how that leads to $\varDelta (X)$ being a linear function of probabilities.

We can always select $p_A$ to be a parameter defined in a chance node not having any chance node descendants (e.g. take $p_A$ to be $p_6$ in the exemplary tree in Fig. 9). Then $p_A$ can be multiplied by any other probability at maximum in one element of the rearranged sum of products defined above (e.g. $p_6$ is multiplied once by $p_3$ and once by $p_5$, and not at all by $p_2$ or $p_4$). As the cross partial derivative is zero, then the whole product must be equal to zero at *DT* (due to the properness the zeroing must happen via zero constant parameters rather than other probabilities in the product being equal to zero). Thus we can remove from our sum of products all the elements containing probabilities defined in the chance nodes farthest from the root (not having chance node descendants). But then the probabilities defined for the second to last chance nodes can only appear in at most one product, and so can be removed (e.g. having removed $-10p_3p_5p_6$, now $p_5$ only appears in one product, i.e. $-10p_3p_5$). Proceeding recursively we can remove all the terms containing two probabilities or more and end up with a linear function of probabilities. The remaining two cases also need to be analyzed.

If no probabilities are left in the linear function, i.e. $\varDelta (X)$ is trivially a constant function, then it must be that $\varDelta (DT)=0$ and so $\varDelta (\cdot )\equiv 0$, which would contradict $\varDelta (DT')>0$. Hence, there must be some probabilities left in the linear function. We can then use properness to see that any of these probabilities can be slightly perturbed to increase or decrease $\varDelta (X)$, and so *DT* is indeed no extremum. $\square$

### Proof (of Remark 4)

We can rewrite the optimization problem given by Eq. (8) as:

$$\begin{aligned} \begin{aligned}&\text {minimize } \quad \sum _{i=1}^{n}x_i\ln \left( x_i/p_i\right) \\&\text {subject to:}\quad \sum _{i=1}^{n}x_i\ln \left( nx_i\right) =\theta \,\wedge \, \sum _{i=1}^nx_i=1\,\wedge \, x_i\ge 0. \end{aligned} \end{aligned}$$

We can discard non-negativity constraints, as we are working with logarithms of $x_i$. If we deal with improper trees, then, for the Kullback–Leibler divergence to be defined, we have to fix $x_i=0$ for such *i* that $p_i=0$. Therefore we can solve the above problem using a system of Lagrangean equations:

$$\begin{aligned} \begin{aligned} \sum _{i=1}^{n}x_i\ln \left( nx_i\right) =\theta \wedge \sum _{i=1}^nx_i=1 \wedge \forall i:\ln (x_i/p_i) + 1 = \lambda _1(\ln (nx_i) + 1) + \lambda _2. \end{aligned} \end{aligned}$$

If $\forall i\, p_i=n^{-1}$ (a degenerate case that starting distribution is equal to the uniform one), then $\lambda _1=1$, $\lambda _2=2\ln (n)$ and all admissible $x_i$ are equally good.

If $p_i$s are not constant, then $\lambda _1\ne 1$ and:

$$\begin{aligned} \begin{aligned}&\sum _{i=1}^{n}x_i\ln \left( nx_i\right) =\theta \wedge \sum _{i=1}^nx_i=1\\&\forall i: x_i = Ap_i^B \wedge A=\exp \left( \frac{1-\lambda _1(1+\log (n))-\lambda _2}{1-\lambda _1}\right) \wedge B=1/(1-\lambda _1), \end{aligned} \end{aligned}$$

where $\lambda _1$ and $\lambda _2$ are determined using the constraints. A solution always exists when $\sum _{i=1}^{n}x_i\ln \left( nx_i\right) =\theta$ can be satisfied, i.e. for $\theta \in [0,\ln (n)[$, and this maps to non-negative values of *B* (*A* is a normalizing constant). This means that in Eq. (9) $\gamma$ can take any non-negative value. $\square$

### Proof (of Remark 5)

*d* is *P*-optimal for any $DT'$, $||DT,DT'||_s\le I(DT,d,s)$.

1. Take specifically $DT'$ which minimizes $P(DT',d)$. Observe that *d*’s expected payoff is greater than or equal to the expected payoffs of other decisions for $DT'$, and so and so must be greater than or equal to the expected payoffs of every other decision for its most unfavorable perturbation.
2. For any other decision, take specifically $DT'$ that maximizes this decision expected payoff. Still, *d*’s expected payoff is greater or equal, and so *d*’s most favorable perturbation must offer greater or equal expected payoff.
3. Selecting a mode-tending perturbation does not depend on the specific decision under consideration; hence, for this mode-tending perturbation *d* will be surely *P*-optimal. $\square$

## Footnotes

1 Using function *d* defined for a larger tree *DT* also for subtrees, e.g. ${DT(e_2)}$, causes no problems.
